# Improved Q-Learning Algorithm Based on Approximate State Matching in Agricultural Plant Protection Environment

**DOI:** 10.3390/e23060737

**Published:** 2021-06-11

**Authors:** Fengjie Sun, Xianchang Wang, Rui Zhang

**Affiliations:** 1 College of Computer Science and Technology, Jilin University, Changchun 130012, China; sunfj17@mails.jlu.edu.cn (F.S.); xcwang89@jlu.edu.cn (X.W.); 2 Key Laboratory of Symbolic Computing and Knowledge Engineering of Ministry of Education, Jinlin University, Changchun 130012, China; 3 Chengdu Kestrel Artificial Intelligence Institute, Chengdu 610000, China

**Keywords:** decision-making support system, reinforcement learning, Q-learning

## Abstract

An Unmanned Aerial Vehicle (UAV) can greatly reduce manpower in the agricultural plant protection such as watering, sowing, and pesticide spraying. It is essential to develop a Decision-making Support System (DSS) for UAVs to help them choose the correct action in states according to the policy. In an unknown environment, the method of formulating rules for UAVs to help them choose actions is not applicable, and it is a feasible solution to obtain the optimal policy through reinforcement learning. However, experiments show that the existing reinforcement learning algorithms cannot get the optimal policy for a UAV in the agricultural plant protection environment. In this work we propose an improved Q-learning algorithm based on similar state matching, and we prove theoretically that there has a greater probability for UAV choosing the optimal action according to the policy learned by the algorithm we proposed than the classic Q-learning algorithm in the agricultural plant protection environment. This proposed algorithm is implemented and tested on datasets that are evenly distributed based on real UAV parameters and real farm information. The performance evaluation of the algorithm is discussed in detail. Experimental results show that the algorithm we proposed can efficiently learn the optimal policy for UAVs in the agricultural plant protection environment.

## 1. Introduction

The Decision-making Support System (DSS) is an important subject in Multi-agent Systems (MAS), where agents select action in each state according to the policy, and it has been gaining considerable attention due to its applications in entertainment, education, traffic, and urban engineering. The policy can be obtained through rule-making or through learning. Reinforcement Learning (RL) has gained great successes in many areas, however, traditional reinforcement learning methods, such as Q-learning [1] and Policy Gradient Learning [2], are poorly suitable in an environment with continuous and high dimensional states and actions [3]. Recently, Deep Reinforcement Learning (DeepRL) [4] has gained remarkable achievements in many research areas such as physics-based animation, robotics, computer vision, and games. It aims at finding an optimal policy that maximizes cumulative rewards and therefore is quite suitable for solving problems with continuous and high dimensional states and actions [5]. However, DeepRL also has its limitations [6]: (1) Parameters are difficult to determine; (2) lack of scalability; and (3) long training time. Therefore, we need to find specific solutions to specific problems.

A reality of the agricultural plant protection environment is the decision-making problem for Unmanned Aerial Vehicles (UAV). With the accelerated development of industrialization and urbanization, agricultural labor shortages have caused labor costs to rise sharply. There are approximately 2 billion hectares of arable land in the world, dozens of major pests and diseases occur all year round, and a considerable amount of agricultural plant protection work needs to be completed. According to statistics from the World Health Organization (WHO) in recent years, more than 3 million cases of pesticide poisoning occur every year around the world [7]. With advancement in technologies, UAV can be useful in agricultural plant protection for crops to execute multiple tasks, such as watering and spraying pesticides. There has been some research focus on UAV path planning in agricultural plant protection environment [8] and UAV spray field evaluation [9,10]. Specifically, UAVs should reach the position of the crops to spray pesticides or water, and return to the base station to supplement energy when the energy is insufficient. To get the optimal policy for UAVs in agricultural plant protection environment, various real situations need to be considered. As UAVs cannot determine the specific position of crops at the beginning, the method of formulating norms cannot be applied in this environment. In this work, we make an assumption that the infrastructure of the plant protection environment is very poor which is realistic in some underdeveloped areas. Specifically, the exact value of the remaining energy of UAVs cannot be transmitted to the commander and the position of UAVs cannot be determined by GPS in real time. Some researchers use the history of actions executed by the agent as an aid for the next step. In [11], the socially intelligent personal agent uses the history of acting and evaluating actions to choose a plan. In [12], agents use the record of actions’ past performance and serves as the basis for making decisions. In this work, we take the history record of UAVs’ actions as the current state, this leads to different states possibly having different lengths, and through experiments we find that the classic Q-learning algorithm and DeepRL algorithm are not suitable for solving problems in this environment.

In this work, we study the problem of forming a policy for UAVs through reinforcement learning in agricultural plant protection environment, which is used as an example to conduct research, and the model and conclusions obtained can be applied to other decision-making or reinforcement learning problems. The main contributions of this paper are as follows:

1. Designing a reinforcement learning model for UAV pesticide spraying in the agricultural plant protection environment;
2. Proposing an Approximate State Matching Q-learning algorithm to solve the policy learning problem in the agricultural plant protection environment;
3. Analyzing the performance of the algorithm and explain the reason that it performs better than the classic Q-learning algorithm in the form of theorems;
4. Testing and evaluating the performance of the proposed method on datasets generated based on real agricultural plant protection environment.

The remainder of the paper is structured as follows. Section 2 presents the literature survey. Section 3 explains the problem and detailed model of the decision-making system in a agricultural plant protection environment. Section 4 describes the key elements involved in the implementation of algorithms we proposed and analyze the properties of the algorithm in detail. Section 5 discusses experiments and results of the implemented methodology. Section 6 concludes the findings of this research propose future work.

## 2. Background

The focus of this work is to make the UAVs learn an optimal policy successfully in an unknown agricultural plant protection environment, which can be formalized as a RL problem. In this section, we first introduce the current research status of related issues, and then briefly introduce reinforcement learning.

### 2.1. Related Work

There are already some studies on the problems related to the use of UAVs in agricultural plant protection. The researchers in [13] proposed a path planning algorithm with the goal of minimizing the total energy consumption of the work, realizing the full coverage path planning of the field and the optimization of the return point position. The researchers in [14] proposed a path planning method based on the Grid-GSA algorithm, which can plan a reasonable return point for the field with irregular boundaries, which makes the non-plant protection operation time the shortest. The researchers in [15] fully consider the influence of the additional flight distance caused by the change of flight height, and studied a path planning method for plant protection UAVs that can be used for a three-dimensional terrain. However these works did not take into account the differences between plant protection tasks. The researchers in [8] propose an method to solve the problem of fairly allocating tasks in an agricultural plant protection environment and consider the situation that the cost of tasks is multiple dimensions. The researchers in [16] use swarm intelligence algorithms to solve the problem of scheduling and route planning for UAV in an agricultural plant protection environment, which is a NP-complete problem. However, none of these works research the policy of a UAV choosing actions based on real-time state.

The researchers in [17] contributes to the summarization of the Conflict Detection and Resolution (CDR) methods and several typical characterizations of the taxonomy have been utilized to articulate their basic functions. The researchers in [18] proposed a causal encounter model to extend the TCAS logic considering the horizontal resolution manoeuvres, which not only provides a better comprehension of the potential collision occurrences for risk assessment by representing the cause-effect relationship of each action, but also aids the pilots in the involved aircraft to make a cooperative and optimal option. These works have greatly inspired our research, however, they did not explore the agricultural plant protection environment.

Policy can be formulated through norms. Norms have multiple manifestations in multi-agent systems, such as obligations, approvals, promises, and prohibition of social norms [11,19]. The coordination effect of norms on agents is to create obligatory behaviors and prohibit behaviors as well as other types of behaviors through a single agent. Obligation norms require agents to perform specific behaviors, while prohibition norms require agents not to perform specific behaviors [20]. In norm-aware practical reasoning, a number of different methods for reasoning about norm compliance have been proposed [21]. The researchers in [22] describe algorithms that enable agents to react to the activation and expiration of norms by modifying their intentions. The researchers in [23] discuss the introduction of a preference relation over norms to solve normative conflicts. This preference relation is taken into account only in situations where it is not possible to comply with all norms. The researchers in [24] use Markov Decision Processes (MDPs) to model a self-interested agent that takes into account norms, and the possibility of violating them, in deciding how to act. The researchers in [21] present a novel multi-agent planning model, Normative Decentralized Partially Observable Markov Decision Processes, and associated heuristic, Most-Critical-States, which can compute effective joint plans given a qualitative reward function. An agent may face conflicts between multiple applicable norms [25], or between norms and its own goals. The researchers in [26] develop a norm compliance framework to design socially adaptive agents in which agents identify and adopt new norms, and determine execution mechanisms to comply with those norms. The researchers in [11] study the problem of whether to reveal its context to others when it deviates from a norm as a socially intelligent personal agent. However, as the specific position of crops and the exact value of the remaining energy of UAVs is unknown at the beginning, it is difficult to formulate effective and stable norms for UAVs in an agricultural plant protection environment.

Policy can be obtained through Reinforcement Learning (RL), with the Q-learning algorithm [1,27] being the most popular classical RL algorithm. The main idea of such methods is to make agents find an optimal control policy that maximizes the reward received in the long term by interacting with the environment by trial and error [5]. The Q-learning algorithm has been used to solve the mobile robots [4,28], pedestrian navigation [29], crowd simulation [30], path planning [31], and other application problems. Some works have also tried to improve the classic Q-learning algorithm. The researchers in [32] proposed a heuristic function to accelerate the algorithm in finding the optimal policy. The researchers in [33] proposed a modified version of the Frequency Maximum Q value heuristic to optimize the problem that the classical Q-learning algorithm does not perform well in stochastic games. The Inverse Reinforcement Learning (IRL) [34] is another classical approach widely used, which refers to an algorithm that reversely derives the reward function of Markov decision process under the premise of a given policy or some operational demonstrations, and allows agents to learn how to make complex problems through expert trajectories. The researchers in [35] used IRL to learn human-like navigation behavior based on example paths. However, the Q-learning algorithm and IRL are mostly used in discrete environments. Deep Reinforcement Learning (DRL) has provided new tools in dealing with high-dimensional continuous control problems [5]. A Deep Q Network (DQN) is a famous DRL algorithm that combines the Q-learning algorithm and neural networks in order to make better training stability and convergence [36]. Another algorithm used widly is Deep Deterministic Policy Gradient (DDPG), which uses a network to fit the policy function in terms of action output and directly outputs actions, coping with the output of continuous actions and a large action space [37]. The DRL algorithms are used to solve problems in various environments. The researchers in [5] uses Proximal Policy Optimization (PPO) [38] combined with Long Short-Term Memory (LSTM) [39] to solve crowd navigation problems in a dynamic environment. The researchers in [3] proposed a Multi-Agent Deep Deterministic Policy Gradient (MADDPG) and used it to solve a multi-UAV combat task problem. The DQN algorithm was adopted to realize cooperative spectrum sensing in cognitive radio networks in [40]. In [41], a Reinforcement Learning-based symbol synchronizer is proposed and a proved valid timing recovery is proven. However, none of these work considered using the history of actions executed by the agent as an aid for the next step. Moreover, we did not use DRL for the following reasons.

1. The DRL algorithm may take longer than the traditional Q learning algorithm, and DRL may require better computer equipment;
2. DRL includes many and complex neural network structures. The optimization of neural networks is a difficult problem. It is possible that a small structural change may cause the experimental results to vary greatly, and it is difficult for us to know the reason for it.

In [11], the socially intelligent personal agent uses the history of acting and evaluating actions to choose a plan. In [12], agents use the record of actions’ past performance to assist in policy optimization and choose individual actions and joint actions using the Q-learning algorithm. However, the methods mentioned above used other information (e.g., position coordinate) as an aid in the process of policy learning. In this work, we study a situation that learning the optimal policy for UAV in an agricultural plant protection environment and the history record of executed actions is the only information that can be used. The existing algorithm does not perform well when solving this problem and this is because the state space in this work is large and different states may have different lengths.

### 2.2. Reinforcement Learning

The goal of the agent in a RL problem is to learn an action policy that maximizes the expected long term sum of values of the reinforcement signal, from any starting state [32]. A policy π: S→A is some function that tells the agent which actions should be chosen and under which circumstances [42]. This problem can be formulated as a discrete time, finite state, and finite action Markov Decision Process (MDP), since problems with delayed reinforcement are well modeled as MDPs [32]. The learner’s environment E∈E can be modeled [43] by a 4-tuple <S,A,T,R>, where:

-S: Is a finite set of states;

-A: Is a finite set of actions that the agent can perform;

-T:S×A:→Π(S): is a state transition function, where Π(S) is a probability distribution over S. $T(s,a,s^{\prime })$ represents the probability of moving from state *s* to $s^{\prime }$ by performing action *a*.

-R:S×A:→R: Is a scalar reward function.

The task of a RL agent is to learn an optimal policy $\pi^{*}:S\to A$ that maps the current state *s* into a desirable action *a* to be performed in *s*. In RL, the policy π should be learned through trial-and-error interactions of the agent with its environment, that is, the RL learner must explicitly explore its environment.

The Q-learning algorithm which was proposed by [27] is a classic algorithm to solve the problem of reinforcement learning. The goal of a Q-learning algorithm is to learn the Q-function, and the optimal policy $\pi^{*}:S\to A$ that maps the current state *s* into an action *a* to get the largest global reward is inferred from the Q-function. The general process of the algorithm has been described in Algorithm 1.
**Algorithm 1** Q–Learning Algorithm**Input:** State set *S*, Action set *A*, Reward function *R*
**Output:**
*Q* table
  1: Initialize *Q* table
  2: **while**
i≤ the number of iterations **do**
  3:    Return *s* to the initial state
  4:    **while** *s* is not terminal **do**
  5:        Choose *a* from *A* using policy derived from Q(s) (e.g., ϵ− greedy)
  6:        Take action *a*, observe *r*, $s^{{}^{\prime }}$
  7:        $Q\left(s,a\right)\leftarrow Q\left(s,a\right)+\alpha [r+\gamma max_{a^{{}^{\prime }}}Q\left(s^{{}^{\prime }},a^{{}^{\prime }}\right)-Q\left(s,a\right)]$
  8:        $s\leftarrow s^{{}^{\prime }}$
  9:    **end while**
10: **end while**
11: Return *Q*


In Algorithm 1, *a* is the agent’s chosen action, *r* is the reward received, Q(s,a) is the value of action *a* in state *s*, and α∈[0,1] is the learning rate. γ is the boolean variable and its value is controlled by the reward function. The policy π can be computed according to the *Q* table returned by Algorithm 1, the following two methods are used commonly.

1. Boltzman distribution:

(1) $$\forall a\in A,\pi \left(s,a\right)=P\left(\underline{a}=a|s\right)$$

(2) $$P\left(\underline{a}=a|s\right)=\frac{e^{\frac{Q(s,a)}{ℵ}}}{\sum_{u\in A}e^{\frac{Q(s,u)}{ℵ}}}$$

where *ℵ* is the temperature parameter that decreases the amount of randomness as it approaches zero.

2. ϵ−greedy policy: In this policy, an agent chooses the best action which has the biggest Q(s,a) in state *s* with probability (1−ϵ) (exploitation mode), and otherwise selects a uniformly random action with probability ϵ (exploration mode).

## 3. Problem Description

In this section, we will formally propose the problem model. In the following models and algorithms, we will encounter some symbols. In order to facilitate understanding, we briefly summarize the implication of the main symbols in Table 1, and the precise definition of the symbols will be described in detail in the following. The focus of this work is to make UAVs learn an optimal policy successfully in an unknown agricultural plant protection environment. In this environment, UAV should fly to the position of the crops and spray pesticides. However in the initial stage, UAVs do not know the exact position of the crop, the amount of pesticide required by the crop, and its own energy reserves. The specific environment representation $E=<S_{u},A_{u},T_{u},R_{u}>$ is shown in the following.

### 3.1. State

As is shown in Section 1, in this work we take the history of UAVs’s actions as the current state *s* of UAVs and s=∅ in the initial state. Such states make up the state space $S_{u}$ which is used to learn the optimal policy in the algorithm. The state contains UAVs’ position information, energy cost, and task completion status. Using the history of actions as the current state is based on the following 3 advantages:

(1) The history of actions includes not only the information of position, but also other information such as energy cost. For instance, [f,f,f,b,b] and [f,f,b] have the same position but different energy cost.
(2) Since in the initial state, the UAV does not know the exact position of the mission, it is necessary to add a dimension to record where the UAV has been sprayed with pesticides, but this will cause the convergence rate to be greatly slowed down. Using the history of actions can solve this problem, because in the learning process, only the state of spraying pesticides at the correct position will be rewarded.
(3) Only considering the position state may lead to the wrong cycle in the learning process, for instance, if the UAV can get a reward for returning to the base station, it will continue to execute the action of returning to the base station. In addition, using the history of actions as an aid can avoid this situation.
(4) Using the history of actions as a state space can be seen as the limited expansion of using coordinates as state space. This is because the action spray pesticide (*s*) and supplementary energy (*e*) are a special action. For instance, [f,f,e,f,f] and [f,f] are (2,2), but the advantages and disadvantages of the two in action selection are obviously different. Similarly, [f,f,s,f,f] and [f,f,f,s,f] are (4,3), but their actual effects may be the same (both sprayed in the wrong position) or different (one sprayed in the correct position). For these actions, it is not enough to add a dimension to record whether they are executed. We also need to know where and when these actions are executed. Thus, using the history of actions as a state space can help us find the optimal policy.
(5) Such state space expansion seems infinite, to solve this problem we will propose the Approximate State Matching Q-learning Algorithm in Section 4.1.
(6) Of course, we can also find the optimal strategy by increasing the number of iterations and randomness. However, this situation can only be verified through experiments, but it is difficult to analyze theoretically. In this work, we are committed to improving the traditional Q learning algorithm and explore theoretically why the improved algorithm is better than the traditional algorithm, as we did in Section 4.2.

**Definition** **1.** *(Length of state)*

*The length of state s is expressed as len(s) which means the number of all actions executed from the initial state to state s.*

### 3.2. Actions

As is shown in Section 2.1, past works have focused on solving the problem of UAV path planning in the agricultural plant protection environment; in this work we focus on optimal policy learning. Six actions are considered in this work: Forward (*f*), Back (*b*), Left (*l*), Right (*r*), Spray Pesticides (*p*), and Supplement Energy (*e*). Thus, the action space $A_{u}=\left\{f,b,l,r,p,e\right\}$. The specific implications of the actions are described in detail in Table 2.

**Definition** **2.** *(Count)*

*The number of occurrences of action a in state s is expressed as count(s,a).*

For instance, we assume s=[f,f,p,e,f,f,p] then count(s,f)=4.

**Definition** **3.** *(The position of action in state)*

*The position of action a in state s is expressed as $\vec{P}\left(s,a\right)$, which means all positions of action a in state s. And the maximum position is expressed as $|\vec{P}{\left(s,a\right)|}_{max}$.*

For instance, we assume s=[f,f,p,e,f,f,p] then the position of action *f* in state *s* is $\vec{P}\left(s,f\right)=\left[0,1,4,5\right]$, and the maximum position $|\vec{P}{\left(s,f\right)|}_{max}=5$.

**Definition** **4.** *(Substate)*

*The substates of state s is expressed as $sub(s,pos_{1},pos_{2})$ which means capture actions in s from $pos_{1}$ to $pos_{2}$.*

It is worth noting that $pos_{1}$ is included but $pos_{2}$ is not included in the substate. For instance, we assume s=[f,f,p,e,f,f,p] then sub(s,0,3) is [f,f,p].

### 3.3. Transition

The environment in this work is taking the history of actions executed by UAVs into the current state of UAVs, the following are the 4 basic rules of state transition.

1. In general, the transition fuction $s^{\prime }=T_{u}\left(s,a\right)$ of state is to add the executed action to the current state.
2. In order to indicate the number of tasks that UAVs have completed in the current state, we set $s_{d}\in Z$ for each state *s*. $s_{d}$ represents the number of tasks that have been completed in state *s*. Thus, in the initial state, $s_{d}=0$, when a task is completed $s_{d}=s_{d}+1$ and $s_{d}$ is added to state *s*.
3. If all tasks are completed, the state will transition to the end state.
4. If the UAV runs out of energy, the state will clear all actions but keep $s_{d}$ to record the number of tasks that have been completed.

### 3.4. Reward

In reinforcement learning, the reward function, $R(s,a,s^{\prime })$, is used as a training signal to encourage or discourage behaviors [5]. The reward function provides a scalar value reflecting the desirability of a particular state transition that is observed by performing an action starting in the initial state *s* and resulting in a successor state $s^{\prime }$ [5].

Since the setting of reward function in this work is closely related to the agricultural plant protection environment, we need to present details of the agricultural plant protection environment which is a complicated problem. UAVs considered in this work are special identical agents for agricultural plant protection, which can handle agricultural plant protection tasks. The integral factors of the problem are defined as follows.

1. UAV starts from the base station, arrives at the position of plant protection tasks, and sprays pesticides.
2. The position and pesticides required of different tasks are different.
3. When the energy is insufficient, the UAV needs to return to supplement.
4. The base station is the end point of Back (*b*), and the position no longer changes if *b* continues to be executed.
5. When executing action *e*, UAVs will return to the position of the base station and replenishes energy.
6. After it completes a task, the UAV will execute the next task that is assigned.
7. When UAVs have multiple tasks, they need to be executed sequentially (i.e., UAVs can not skip the current task and execute the next one).

Based on the above constraints, we define reward function $R_{u}$ on Definition 5.

**Definition** **5.** *(Reward function)*

*For any state $s\in S_{u}$, $a\in A_{u}$ is the action executed in the state s, and $s^{\prime }=T_{u}\left(s,a\right)$; then the reward function:*

$$R_{u}\left(s,a,s^{\prime }\right)=\left\{\begin{array}{cc} r=1;\gamma =0 & \mathrm{when}\;a\;\mathrm{task}\;\mathrm{is}\;\mathrm{completed}\\ r=0;\gamma =0 & e\;\mathrm{is}\;\mathrm{executed}\;\mathrm{when}\;\mathrm{energy}\;\mathrm{is}\;\mathrm{insufficient}\\ r=-1;\gamma =0 & e\;\mathrm{is}\;\mathrm{not}\;\mathrm{executed}\;\mathrm{when}\;\mathrm{energy}\;\mathrm{is}\;\mathrm{insufficient}\\ r=0;\gamma =1 & \mathrm{other}\;\mathrm{situations}. \end{array}\right.$$

## 4. Problem Solution

In this section, we will propose a solution for the problem proposed in Section 3. To address this problem, we will propose an Approximate State Matching Q-learning algorithm to learn the optimal policy in an agricultural plant protection environment. In Section 4.2, we theoretically demonstrate its advantages over classic Q-learning algorithms.

### 4.1. Approximate State Matching Q-Learning Algorithm

As previously shown, due to state space $S_{u}$ approaching infinity, the classic Q-learning algorithm will randomly select actions when encountering unfamiliar states and random selection of actions will produce more unfamiliar states. Therefore, the optimal policy can not be learned by the classic Q-learning algorithm in the environment $E=<S_{u},A_{u},T_{u},R_{u}>$.

Our solution to the unfamiliar state is using the information of its similar state to assist in choosing actions. We select similar states through two key points: Position and energy cost. As described in Section 3, state is the history of actions that can be represented as a queue. Given a state $s\in S_{u}$, Algorithm 2 shows selecting similar set $S_{ene}$ of *s* through energy cost.
**Algorithm 2** Selecting a Similar Set Based on Energy Cost**Input:** States *s*
**Output:** Similar set $S_{ene}$ of *s*
  1: Create a empty stack 
  2: **while**
s≠∅
**do**
  3:    *a* is the action that pops from the head of state *s*
  4:    **if** a=e **then**
  5:        Clear the stack
  6:    **else**
  7:        Push *a* to the stack
  8:    **end if**
  9: **end while**
10: Measure the length of the stack and mark it as *l*
11: $S_{ene}=\emptyset$
12: $S^{\prime }=S_{pa}$
13: **while**
$\left|S^{\prime }\right|\geq \theta \cdot \left|S_{pa}\right|$
 **do**
14:    Select $s^{\prime }$ from $S^{\prime }$
15:    $l^{\prime }=$ executing Line 1 to Line 10 with $s^{\prime }$
16:    **if** $l^{\prime }=l$ **then**
17:        $S_{ene}=S_{ene}\cup \left\{s^{\prime }\right\}$
18:        $S^{\prime }=S^{\prime }\setminus \left\{s^{\prime }\right\}$
19:    **else**
20:        $S^{\prime }=S^{\prime }\setminus \left\{s^{\prime }\right\}$
21:    **end if**
22: **end while**
23: Return $S_{ene}$


In the algorithm, $S_{ene}$ is the set with the same energy cost as state *s*, $S_{pa}$ is the set of previous states, and θ is the exploration rate that can be set. Line 1 to Line 10 of Algorithm 2 calculates the energy cost of UAV in state $s_{1}$. According to the environment $E=<S_{u},A_{u},T_{u},R_{u}>$ described in Section 3, the action *f*, *b*, *l*, *r*, and *p* cost 1 unit of energy and action *e* replenishes the energy, so action *f*, *b*, *l*, *r*, and *p* push to the stack and the stack can be cleared when encountering action *e*. Line 11 to Line 22 of Algorithm 2 look for a similar set $S_{ene}$ of *s* in all passed states $S_{pa}$ according to the energy cost calculated. It is worth noting that as more and more states are passed, the $S_{pa}$ will become larger, which may increase the running time of the algorithm. Our solution to this is to set the θ (0<θ≤1) function on $S_{pa}$. That is, the state of looking does not exceed (1−θ) percent of $S_{pa}$. When the passed states $S_{pa}$ is too large, we can reduce the running time of the algorithm by adjusting θ. Similar to Algorithm 2, Algorithm 3 shows selecting similar set $S_{vp}$ of *s* through vertical position.
**Algorithm 3** Selecting a Similar Set Based on Vertical Position**Input:** States *s*
**Output:** Similar set $S_{vp}$ of *s*
  1: Create 2 empty stack: $stack_{1}$, $stack_{2}$
  2: **while**
s≠∅
 **do**
  3:    *a* is the action that pops from the head of state *s*
  4:    **if** a=f **then**
  5:        Push *a* to $stack_{1}$
  6:    **else if** a=b **then**
  7:        **if** stack≠∅ **then**
  8:           Pop an element from $stack_{1}$
  9:        **end if**
10:    **else if** a=e **then**
11:        Clear $stack_{1}$
12:    **else if** $a=s_{d}$ **then**
13:        Push *a* to $stack_{2}$ and clear $stack_{1}$
14:    **end if**
15: **end while**
16: Measure the length of the $stack_{1}$ and $stack_{2}$ and mark them as $l_{1}$ and $l_{2}$ respectively
17: $S_{vp}=\emptyset$
18: $S^{\prime }=S_{pa}$
19: **while**
$\left|S^{\prime }\right|\geq \theta \cdot \left|S_{pa}\right|$
 **do**
20:    Select $s^{\prime }$ from $S^{\prime }$
21:    Calculate $l_{1}^{\prime }$ and $l_{2}^{\prime }$ by executing Line 1 to Line 16 with $s^{\prime }$
22:    **if** $l_{1}^{\prime }=l_{1}$ and $l_{2}^{\prime }=l_{2}$ **then**
23:        $S_{vp}=S_{vp}\cup \left\{s^{\prime }\right\}$
24:        $S^{\prime }=S^{\prime }\setminus \left\{s^{\prime }\right\}$
25:    **else**
26:        $S^{\prime }=S^{\prime }\setminus \left\{s^{\prime }\right\}$
27:    **end if**
28: **end while**
29: Return $S_{vp}$


In the algorithm, $S_{vp}$ is the set with the same vertical position as state *s*, and $S_{pa}$ is the set of previous states and θ is the exploration rate that can be set. Line 1 to Line 16 of Algorithm 3 is calculating the position of UAV in state *s*. According to the environment $E=<S_{u},A_{u},T_{u},R_{u}>$ described in Section 3, the action *f* means go forward one step, action *b* means go back one step, and action *e* returns to the base station, so that action *f* is pushed to $stack_{1}$, the $stack_{1}$ pop one *f* when encounter action *b* and the $stack_{1}$ be cleared when encounter action *e*. The symbol $s_{d}$ indicates that a task is completed and UAV will come to a new starting position, so the current position status will be cleared and $s_{d}$ is pushed to $stack_{2}$ to record the number of tasks completed. Line 17 to Line 28 of Algorithm 3 is looking for a similar set $S_{vp}$ of *s* in all passed states $S_{pa}$ according to the position calculated.

In Algorithm 3, we only considered the vertical position (i.e., forward, back), not the horizontal position (i.e., left, right). This is because the principle of horizontal position is exactly the same as that of the vertical position. Therefore, in the same way, we can get $S_{hp}$ as the set with the same horizontal position as state *s*.

To better illustrate the Approximate State Matching Q-learning algorithm proposed in this work, the definition of the Guided State is explained.

**Definition** **6.** *(Guided State)*

*If for state s,*

∃a∈A,Q(s,a)≠0

*then state s is called the Guided State. On the contrary, if:*

∀a∈A,Q(s,a)=0

*then state s is not the Guided State.*

Since Q(s,a) changes with the execution of the algorithm, *s* may become a Guided State. In this work, the Q(s,a) of an unpassed state *s* are set to 0 for all a∈A when it is initialized. Thus, all unpassed states are not the Guided State. Table 3 is a simple example of a *Q* table. Next, we will formally propose our Approximate State Matching Q-learning algorithm which is described in detail in Algorithm 4.
**Algorithm 4** Approximate State Matching Q-learning Algorithm**Input:** Environment $E:S=S_{u};\;A=A_{u};\;T=T_{u};\;R=R_{u}$
**Output:** Learned *Q* table
  1: Initialize *Q* table
  2: **while**i≤ the number of iterations **do**
  3:    Return *s* to the initial state
  4:    **while** *s* is not terminal state **do**
  5:        **if** *s* is not in *Q* table **then**
  6:           Add *s* to the *Q* table and initialize *s*
  7:        **end if**
  8:        **if** *s* is a Guided State **then**
  9:           Choose *a* from *A* using policy derived from Q(s)
10:        **else**
11:           Select the similar set $S_{ene}$ of *s* using Algorithm 2
12:           $Q\left(s,a,temporary\right)=\frac{\sum_{\epsilon \in S_{ene}}Q\left(\epsilon ,a\right)}{∥S_{ene}∥}$ for all a∈A
13:           Choose *a* from *A* using policy derived from Q(s,temporary)
14:           **if** a≠e **then**
15:               Select the similar set $S_{vp}\cap S_{hp}$ of *s* using Algorithm 3
16:               $Q\left(s,a,temporary\right)=\frac{\sum_{\left(\epsilon \in S_{vp}\cap S_{hp}\right)\wedge Q\left(\epsilon ,a\right)\geq 0}Q\left(\epsilon ,a\right)}{∥S_{vp}\cap S_{hp}∥}$ for all a∈A
17:               Choose *a* from *A* using policy derived from Q(s,temporary)
18:           **end if**
19:        **end if**
20:        Take action *a*, observe $s^{{}^{\prime }}=T_{u}\left(s,a\right)$ and $r=R_{u}\left(s,a,s^{\prime }\right)$
21:        $Q\left(s,a\right)\leftarrow Q\left(s,a\right)+\alpha [r+\gamma max_{a^{{}^{\prime }}}Q\left(s^{{}^{\prime }},a^{{}^{\prime }}\right)-Q\left(s,a\right)]$
22:        $s\leftarrow s^{{}^{\prime }}$
23:    **end while**
24: **end while**
25: Return *Q* table


(a) Initialization

Line 1 of Algorithm 4 is to initialize the *Q* table. An empty table is created because there is no state at the beginning. Line 5 to Line 7 is to check whether current state *s* has arrived before, if not, then add *s* to the *Q* table.

(b) Choose action

Line 8 to Line 19 is to choose action and two cases are considered, Q(s,temporary) is the temporary *Q* table constructed to guide the unguided state to make the best decision, and the Q(s,temporary) is destroyed after the action is selected. Case one (Line 8 to Line 9) is the current state *s* and is a Guided State which is defined in Definition 6. In this case, *s* will choose action using policy derived from Q(s). Here the ϵ− greedy is used which is described in Section 2.2. The other case (Line 10 to Line 19) is that the current state *s* is not a Guided State. In this case, we divide it into two steps due to the complexity of the situation. Step one (Line 11 to Line 13) is selecting a similar set $S_{ene}$ of *s* through energy cost to build a temporary *Q* table Q(s,temporary) for *s* and determine whether supplementary energy (*e*) is needed in *s*. If action *e* is not chosen, step two will be triggered (Line 14 to Line 18), in this step the vertical position similar set $S_{vp}$ and horizontal position similar set $S_{hp}$ of *s* are selected to build a temporary *Q* table Q(s,temporary) for *s*, it is worth mentioning that those *Q* values less than 0 will not be added. Finally, action is chosen using policy derived from Q(s,temporary).

(c) Execution action

Line 20 of Algorithm 4 is to execute the chosen action and calculate the reward *r* based on the reward function $R_{u}$ defined in Section 3. α∈[0,1] is the learning rate and γ is the control factor, that is γ=0 when *r* exists and γ=1 when *r* does not exist. Line 21 is to update the *Q* table according to *r*, which is the same as Algorithm 1. Line 22 is to go to the next state and continue to loop.

### 4.2. Analysis of Algorithms

In Section 4.1, we proposed an Approximate State Matching Q-learning algorithm to allocate tasks to learn the optimal policy in an agricultural plant protection environment. In this section, we will analyze the algorithm’s property and theoretically demonstrate its advantages over classic Q-learning algorithms. First of all, we need to emphasize that the principles of vertical position (i.e., forward, back) and horizontal position (i.e., left, right) are exactly the same. Therefore, in this section we only take vertical position as an example for analysis.

**Definition** **7.** *($\vec{Q}$ vector)*

*In the environment:*

$$E=<S,A,T,R>\mathrm{where}\;A=\{a_{1},a_{2},\cdots ,a_{n}\}$$

*then for arbitrary s∈S, the $\vec{Q}$ vector:*

$$\vec{Q}\left(s\right)=\left[\begin{array}{cccc} Q(s,a_{1}) & Q(s,a_{2}) & \cdots & Q(s,a_{n}) \end{array}\right].$$

In this work, according to the environment:

$$E=<S_{u},A_{u},T_{u},R_{u}>$$

set in Section 3, the $\vec{Q}$ vector consists of:

$$\left[\begin{array}{cccc} Q(s,f) & Q(s,b) & Q(s,p) & Q(s,e) \end{array}\right]$$

for all $s\in S_{u}$, which will continue to change as the algorithm runs. In order to measure the optimality of the solution, we also need to define the $\vec{J}$ vector which is described in Definition 8.

**Definition** **8.** *($\vec{J}$ vector)*

*In the environment:*

$$E=<S,A,T,R>where\;A=\{a_{1},a_{2},\cdots ,a_{n}\}$$

*then for arbitrary s∈S, the $\vec{J}$ vector:*

$$\vec{J}\left(s\right)={\left[\begin{array}{cccc} J(s,a_{1}) & J(s,a_{2}) & \cdots & J(s,a_{n}) \end{array}\right]}^{T}$$

*where for arbitrary a∈A:*

$$J\left(s,a\right)=\left\{\begin{array}{cc} 0 & Q(s,a)=0\\ \frac{1}{Q(s,a)} & Q(s,a)\neq 0 \end{array}\right..$$

Similar to Definition 7, the $\vec{J}$ vector consists of:

$$\left[\begin{array}{cccc} J(s,f) & J(s,b) & J(s,p) & J(s,e) \end{array}\right]$$

for all $s\in S_{u}$ when E=$<S_{u},A_{u},T_{u},R_{u}>$ and the $\vec{J}$ vector changes with the $\vec{Q}$ vector. In order to measure the optimality of the solution, we should define the global optimal policy $\pi_{glo}$.

**Definition** **9.** *(Global optimal policy)*

*For ∀s∈S, the global optimal policy $\pi_{glo}\left(s\right)$ is the action to achieve the global objective most effectively in state s.*

Taking the global optimal policy as the measurement standard, we can define an evaluation function to evaluate the current policy.

**Definition** **10.** *(Evaluation function)*

*For any state s∈S, a∈A is the action executed in the state s; then the evaluation function:*

$$E\left(s,a\right)=\left\{\begin{array}{cc} 1 & a=\pi_{glo}\left(s\right)\\ -1 & a\neq \pi_{glo}\left(s\right) \end{array}\right..$$

Although Definition 10 is similar to the reward function of Definition 5, but they are fundamentally different. The reward function guides action selection through rewards during the learning process and the evaluation function does not affect the policy but only evaluates the current policy.

**Theorem** **1.** *In environment E=<S,A,T,R> where $S=S_{u},A=A_{u},T=T_{u},R=R_{u}$; and for ∀s∈S, Q(s) and $Q^{\prime }\left(s\right)$ are learned by Algorithm 4. a and $a^{\prime }$ are choosen from A using the ϵ− greedy policy derived from Q(s) and $Q^{\prime }\left(s\right)$, if:*

$${∥\vec{Q}\left(s\right)\cdot \vec{J}\left(s\right)∥}_{1}>{∥\vec{Q^{\prime }}\left(s\right)\cdot \vec{J^{\prime }}\left(s\right)∥}_{1}$$

*then*

$$P\left[E\left(s,a\right)>E\left(s,a^{\prime }\right)\right]>P\left[E\left(s,a^{\prime }\right)>E\left(s,a\right)\right].$$

Theorem 1 shows that there is a higher probability of choosing a more suitable action according to Q(s) than according to $Q^{\prime }\left(s\right)$. Here, the situation in which actions are randomly chosen is not considered (i.e., ϵ=0). In order to prove Theorem 1, we first need to explain some lemmas for ease of explanation.

**Lemma** **1.** *In environment E=<S,A,T,R> where $S=S_{u},A=A_{u},T=T_{u},R=R_{u}$; and for ∀s∈S, Q(s) is learned by Algorithm 4, then:*

∃a∈A,Q(s,a)≥0.

**Proof.**  By contradiction, we assume that Q(s,a)<0 for ∀a∈A, according to Algorithm 4:

(3) $$Q\left(s,a\right)\leftarrow Q\left(s,a\right)+\alpha [r+\gamma max_{a^{{}^{\prime }}}Q\left(s^{{}^{\prime }},a^{{}^{\prime }}\right)-Q\left(s,a\right)].$$

Due to that, Q(s,a)=0 in the initialization state, so:

$$max_{a^{{}^{\prime }}}Q\left(s^{{}^{\prime }},a^{{}^{\prime }}\right)=0.$$

Then r<0 for ∀a∈A, this contradicts “r=−1 when *e* is not executed” in Definition 5. □

**Lemma** **2.** *In environment E=<S,A,T,R> where $S=S_{u},A=A_{u},T=T_{u},R=R_{u}$; and for ∀s∈S, Q(s) is learned by Algorithm 4, if:*

Q(s,p)>0

*then,*

$$\pi_{glo}\left(s\right)=e$$

*and*

∀a∈A∖{p},Q(s,a)=0.

**Proof.**  Due to Q(s,p)>0, we can know that UAV has sufficient energy in state *s*, so according to Definition 5, ∀a∈A∖{p},Q(s,a)≥0. In addition, according to the ϵ− greedy policy, ∀a∈A∖{p} will not be choosen if Q(s,p)>0, thus ∀a∈A∖{p},Q(s,a)=0. We can know that UAV has reached the position of the task if Q(s,p)>0 or else the execution of action *p* will not get positive feedback according to Algorithm 4. Thus, the global optimal policy on state *s* is *p*, thus $\pi_{glo}\left(s\right)=e$. □

**Lemma** **3.** *In environment E=<S,A,T,R> where $S=S_{u},A=A_{u},T=T_{u},R=R_{u}$; and for ∀s∈S, Q(s) is learned by Algorithm 4, if:*

Q(s,f)>0

*then*

$$\pi_{glo}\left(s\right)=f$$

*and*

∀a∈A∖{f},Q(s,a)=0.

**Proof.**  Similarly to Lemma 2, due to Q(s,f)>0, we can know that UAV has sufficient energy in state *s*, so according to Definition 5, ∀a∈A∖{f},Q(s,a)≥0. And according to the ϵ− greedy policy, ∀a∈A∖{f} will not be choosen if Q(s,f)>0, thus ∀a∈A∖{p},Q(s,a)=0. We can know that UAV has not reached the position of the task if Q(s,f)>0 or else the execution of action *f* will not get positive feedback according to Algorithm 4. Thus, the global optimal policy on state *s* is *f*, thus $\pi_{glo}\left(s\right)=f$. □

**Lemma** **4.** *In environment E=<S,A,T,R> where $S=S_{u},A=A_{u},T=T_{u},R=R_{u}$; and for ∀s∈S, Q(s) is learned by Algorithm 4, then:*

Q(s,b)≤0.

**Proof.**  By contradiction, we assume that Q(s,b)>0. We will explain it through 3 cases.

Case 1: UAV has not reached the position of the task in state *s*. Due to action *b* not being able to complete a task, according to the Lemma 3, there is a state $s^{\prime }$ with the same position as *s* in previous states if Q(s,b)>0, and $Q(s^{\prime },f)>0$. This is a contradiction because action *b* will not be chosen in state *s* according to Algorithm 4.

Case 2: UAV has reached the position of the task in state *s*. Due to the action *b* can not complete a task, so according to the Lemma 2, there has a state $s^{\prime }$ with the same position as *s* in previous states if Q(s,b)>0, and $Q(s^{\prime },p)>0$. This is contradiction because the action *b* will not be choosen in state *s* according to the Algorithm 4.

Case 3: UAV has exceeded the position of the task in state *s*. In this case if Q(s,b)>0, then there has a state $s^{\prime }$ in the position of the task in previous states that $Q\left(s,b\right)\leftarrow Q\left(s,b\right)+\alpha [r+\gamma Q\left(s^{\prime },p\right)-Q\left(s,a\right)]$ (i.e., *s* may indirectly get feedback from $s^{\prime }$). This is a contradiction because the action *p* will be chosen when the UAV reaches the same position as the $s^{\prime }$ state according to Algorithm 4, and the state *s* will not be reached.

In summary, Q(s,b)≤0 for ∀s∈S. □

**Lemma** **5.** *In environment E=<S,A,T,R> where $S=S_{u},A=A_{u},T=T_{u},R=R_{u}$; and for ∀s∈S, Q(s) is learned by Algorithm 4, then:*

Q(s,e)≡0.

**Proof.**  We will prove this lemma in 2 cases.

Case 1: *e* is executed when energy is insufficient. In this case, according to Definition 5, we know that R=0, so Q(s,e)=0.

Case 2: *e* is executed when energy is sufficient. In this case, we will prove it by contradiction. First, we assume Q(s,e)>0. In the initial state Q(s,e)=0, according to Definition 5, $R_{u}=1$ when a task is completed and a task cannot be completed by action *e* so $max_{a^{\prime }}Q\left(s^{\prime },a^{\prime }\right)>0$ and UAV in its initial position in state $s^{\prime }$. Then it has a state $s^{\prime \prime }$ with the same position as *s* in previous states and $Q(s^{\prime \prime },f)>0$ or $Q(s^{\prime \prime },p)>0$. This is a contradiction because action *e* will not be chosen in state *s* according to Algorithm 4, so Q(s,e)≤0. Next, we assume that Q(s,e)<0, the only possibility is $max_{a^{{}^{\prime }}}Q\left(s^{{}^{\prime }},a^{{}^{\prime }}\right)<0$ according to Definition 5. This conflicts with Lemma 1, so Q(s,e)≥0. In summary, Q(s,e)≡0. □

**Proof.**  Proof of Theorem 1. We will prove this theorem in 3 cases.

Case 1: Q(s,p)>0. In this case:

$${∥\vec{Q}\left(s\right)\cdot \vec{J}\left(s\right)∥}_{1}>{∥\vec{Q^{\prime }}\left(s\right)\cdot \vec{J^{\prime }}\left(s\right)∥}_{1}$$

if and only if $Q^{\prime }\left(s,a\right)=0$ for ∀a∈A according to Lemma 2. And $\pi_{glo}\left(s\right)=p$, so $P[E\left(s,a\right)>E\left(s,a^{\prime }\right)]=3/4$ and $P[E\left(s,a^{\prime }\right)>E\left(s,a\right)]=0$.

Case 2: Q(s,f)>0. This case is the same as case 1, except that it needs to refer to Lemma 3.

Case 3: Q(s,p)≤0 and Q(s,f)≤0. In this case we set that:

$$A^{*}\subset A,\;Q\left(s,a\right)<0\;\mathrm{for}\;\forall a\in A^{*}$$

and

$$A^{\prime }\subset A,\;Q^{\prime }\left(s,a\right)<0\;\mathrm{for}\;\forall a\in A^{\prime }$$

we set $|A^{*}|=m$ and $|A^{\prime }|=n$ according to Lemmas 4 and 5 we can know that 0≤m,n≤3. Then ${∥\vec{Q}\left(s\right)\cdot \vec{J}\left(s\right)∥}_{1}>{∥\vec{Q^{\prime }}\left(s\right)\cdot \vec{J^{\prime }}\left(s\right)∥}_{1}$ if and only if m>n. According to Definition 5 we can know that UAV has insufficient energy in state *s*, so $\pi_{glo}\left(s\right)=e$. Thus $P\left[E\left(s,a\right)>E\left(s,a^{\prime }\right)\right]=\frac{3-n}{\left(4-m)\cdot (4-n\right)}$ and $P\left[E\left(s,a^{\prime }\right)>E\left(s,a\right)\right]=\frac{3-m}{\left(4-m)\cdot (4-n\right)}$. □

Through Theorem 1, we can know that the $\vec{J}$ vector can be used to measure the optimality of the *Q* table. The specific measurement method is to judge whether UAV can choose action *a* which conforms to the global optimal policy in the state *s* using the *Q* table. However under the environment set in Section 3, the probability of *s* is a Guide State is reduced due to state space being too large. Our solution to this is that we propose to establish a temporary *Q* table Q(s,temporary) for the state *s* to assist in the selection of actions, which has been shown in Algorithm 4. Next, we will explain that the optimality of Q(s,temporary) can be improved under a particular environment.

**Lemma** **6.** *In environment E=<S,A,T,R> where $S=S_{u},A=A_{u},T=T_{u},R=R_{u}$; s is not a Guide State and the UAV has insufficient energy in state s; $S_{ene}$ and $S_{ene}^{\prime }$ are a similar set of s based on energy cost; Q(s,temporary) and $Q^{\prime }\left(s,temporary\right)$ are established by $S_{ene}$ and $S_{ene}^{\prime }$ respectively using Algorithm 4, if:*

$$S_{ene}^{\prime }\subset S_{ene}$$

*then*

$$\begin{array}{cc} \; & ∥\vec{Q}\left(s,temporary\right)\cdot \vec{J}{\left(s,temporary\right)∥}_{1}\\ \; & \geq ∥\vec{Q^{\prime }}\left(s,temporary\right)\cdot \vec{J^{\prime }}{\left(s,temporary\right)∥}_{1} \end{array}.$$

**Proof.**  We set the complement set of $S_{ene}^{\prime }$ as $S_{c}$, that is $S_{ene}=S_{ene}^{\prime }\cup S_{c}$ and $S_{ene}^{\prime }\cap S_{c}=\emptyset$. Here we use $\vec{Q}\left(s,tem\right)$ to represent $\vec{Q}\left(s,temporary\right)$ for convenience. Then:

$$\begin{array}{cc} \; & ∥\vec{Q}\left(s,tem\right)\cdot \vec{J}{\left(s,tem\right)∥}_{1}\\ \; & =|\sum_{a\in A}Q\left(s,a,tem\right)\cdot J\left(s,a,tem\right)| \end{array}$$

and

$$\begin{array}{cc} \; & Q(s,a,tem)\cdot J(s,a,tem)\\ \; & =\frac{\sum_{\epsilon \in S_{ene}}Q\left(\epsilon ,a\right)}{|S_{ene}|}\cdot J\left(s,a,tem\right) \end{array}$$

due to the UAV has insufficient energy in state *s* so Q(ϵ,a)≤0, and according to Definition 8, we can get that $∥\vec{Q}\left(s,tem\right)\cdot \vec{J}{\left(s,tem\right)∥}_{1}$ is a negative correlation with $\sum_{\epsilon \in S_{ene}}Q\left(\epsilon ,a\right)$. Due to:

$$\sum_{\epsilon \in S_{ene}}Q\left(\epsilon ,a\right)=\sum_{\epsilon \in S_{ene}^{\prime }\cup S_{c}}Q\left(\epsilon ,a\right)$$

so

$$\sum_{\epsilon \in S_{ene}}Q\left(\epsilon ,a\right)\leq \sum_{\epsilon \in S_{ene}^{\prime }}Q\left(\epsilon ,a\right)$$

thus,

$$\begin{array}{cc} \; & ∥\vec{Q}\left(s,temporary\right)\cdot \vec{J}{\left(s,temporary\right)∥}_{1}\\ \; & \geq ∥\vec{Q^{\prime }}\left(s,temporary\right)\cdot \vec{J^{\prime }}{\left(s,temporary\right)∥}_{1} \end{array}.$$

□

The $S_{ene}$ provides guidance for those that are not a Guide State and have insufficient energy. For those that are not a Guide State and have sufficient energy, $S_{vp}$ is used to assist the selection of action. Lemma 7 explains how to improve optimality in this environment.

**Lemma** **7.** *In environment E=<S,A,T,R> where $S=S_{u},A=A_{u},T=T_{u},R=R_{u}$; s is not a Guide State and the UAV has sufficient energy in state s; $S_{vp}$ and $S_{vp}^{\prime }$ are a similar set of s based on position; Q(s,temporary) and $Q^{\prime }\left(s,temporary\right)$ are established by $S_{vp}$ and $S_{vp}^{\prime }$ respectively using Algorithm 4, if:*

$$S_{vp}^{\prime }\subset S_{vp}$$

*then*

$$\begin{array}{cc} \; & ∥\vec{Q}\left(s,temporary\right)\cdot \vec{J}{\left(s,temporary\right)∥}_{1}\\ \; & \geq ∥\vec{Q^{\prime }}\left(s,temporary\right)\cdot \vec{J^{\prime }}{\left(s,temporary\right)∥}_{1} \end{array}.$$

The proof of Lemma 7 is similar to the proof of Lemma 6. However it is worth mentioning that only $\epsilon \in S_{vp}\wedge Q\left(\epsilon ,a\right)\geq 0$ are used to construct Q(s,temporary), this is because for those of $\epsilon \in S_{vp}\wedge Q\left(\epsilon ,a\right)<0$ have insufficient energy.

From Lemmas 6 and 7, it can be concluded that the larger the similarity sets $S_{ene}$ and $S_{vp}$, the higher the probability of choosing the optimal action in state *s*. As the classic Q-learning algorithms do not construct $S_{ene}$ and $S_{vp}$, we can regard the size as 0. For Algorithm 4, affected by factors such as randomness and number of iterations, the size of $S_{ene}$ and $S_{vp}$ are difficult to determine. Thus, we will measure the algorithm using the theoretical maximum size of the set, which represents the maximum size that the set can theoretically reach.

It is easy to know that the theoretical maximum size of $S_{ene}$ and $S_{vp}$ are infinite theoretically in the environment set in Section 3. Thus, we will explain the theoretical maximum size of $S_{ene}$ and $S_{vp}$ under certain conditions through theorems in the following.

**Theorem** **2.** *In environment E=<S,A,T,R> where $S=S_{u},A=A_{u},T=T_{u},R=R_{u}$; $S_{L}$ is a state set that:*

$$S_{L}=\left\{s|s\in S_{u}\wedge len\left(s\right)\leq L\right\}$$

*the energy cost of the UAV in state s is l; $S_{ene}$ is the similar set of s based on energy cost, then the theoretical maximum size of $S_{L}\cap S_{ene}=\left(3^{L-1}+2\cdot 3^{l-1}\right)$.*

**Proof.**  The first thing we emphasize is that the symbol “$s_{d}$” is not counted in the length of the state because it is not an action, and only represents the completion of a task. As the energy cost of *s* is *l*, the last *l* actions in state *s* do not contain action *e*, thus there are $3^{l}$ situations. The length of states do not exceed *L*, for the previous actions, there are several cases.

Case 1: No action in front.

Case 2: There is one action, then the action must be *e*.

Case 3: There are two actions, then the last action must be *e*.

⋯

Case (L−l+1): There are (L−l) actions, then the last action must be *e*.

Therefore, for the previous action, the total situation is:

$$1+1+4+\cdots +4^{\left(L-l-1\right)}=\frac{3^{\left(L-l\right)}-1}{3}+1.$$

Thus, the theoretical maximum size of $S_{L}\cap S_{ene}=\left(3^{L-1}+2\cdot 3^{l-1}\right)$. □

Theorem 2 has explained the size of $S_{ene}$ in which the length of states do not exceed *L*. For the theoretical maximum size of $S_{vp}$, the situation is more complicated, and we need to make more detailed restrictions in this regard. The following equations of polynomial will be used to assist the explanation of the theorem.

(4) $$\sum_{n=0}^{12}a_{n}\cdot z^{n}=\left(z^{5}+z^{4}+z^{3}+z^{2}+z+1\right)\cdot \left(z^{4}+z^{2}+1\right)\cdot \left(z^{3}+1\right)$$

(5) 1(1−z6)3=∑k≥0k+22z6·k.

In Equation (4),

$$a_{n}=\left[z^{n}\right]\sum_{n=0}^{12}a_{n}\cdot z^{n}$$

that is $a_{n}$ is the coefficient of $z^{n}$ in the polynomial, for instance $a_{0}=1$, $a_{6}=3$, $a_{12}=1$. Equation (5) is derived from:

a(1−p·z)m+1=∑n≥0m+nma·(p·z)n.

**Theorem** **3.** *In environment E=<S,A,T,R> where $S=S_{u},A=A_{u},T=T_{u},R=R_{u}$; $S_{C}$ is a state set that:*

$$S_{C}=\left\{s|cond_{1}\wedge cond_{2}\right\}$$

*where*

$$cond_{1}:s\in S_{u}$$

$$cond_{2}:Cou(sub(s,|\vec{P}{\left(s,e\right)|}_{max},end),f)=C$$

*the advance distance of UAV in state s is d; $S_{vp}$ is the similar set of s based on position, then the theoretical maximum size of SC∩Svp≥∑{(n,k)|6k+n=C−d}an·k+22.*

**Proof.**  As the action *f* has been executed *C* times by UAV, and the advance distance of UAV in state *s* is *d*, we can conclude that C−d times of action *f* is cancelled out by action *b*. For instance, we assume that C−d=2 then the state may contain [f,f,b,b] or [f,b,f,b] but [b,b,f,f] does not meet the condition because there will be no change of position when executing action *b* at the base station. We constructed a generating function to exlpain it. □

For a single action, the generating function is:

(6) $$1+z+z^{2}+z^{3}+\cdots =\frac{1}{1-z}.$$

For a situation where two same actions are connected together, the generating function is:

(7) $$1+z^{2}+z^{4}+z^{6}+\cdots =\frac{1}{1-z^{2}}.$$

For the situation where the three same actions are connected together, the generating function is:

(8) $$1+z^{3}+z^{6}+z^{9}+\cdots =\frac{1}{1-z^{3}}.$$

For the situation where four or more same actions connected together is not calculated because the probability is relatively low, by multipling Equations (6)–(8), we can get:

(9) $$C\left(z\right)=\frac{1}{\left(1-z\right)\left(1-z^{2}\right)\left(1-z^{3}\right)}.$$

By transforming Equation (9), we can get:

(10) $$C\left(z\right)=\frac{\left(z^{5}+z^{4}+z^{3}+z^{2}+z+1\right)\cdot \left(z^{4}+z^{2}+1\right)\cdot \left(z^{3}+1\right)}{{\left(1-z^{6}\right)}^{3}}.$$

According to Equations (4) and (5), the Equation (10) can be expressed as

(11) C(z)=(∑n=012an·zn)·(∑k≥0k+22z6·k)

Then the way of partition is the coefficient of $z^{C-d}$ in C(z), and several situations need to be considered: (1) Position of action *p*; (2) internal sorting of the same partition; (3) action *b* not changing position; and (4) four or more same actions connected together. Thus Theorem 3 is proven.

## 5. Experiment

To examine the behavior and performance of the proposed algorithm, experiments are conducted based on multiple sets of data [13,14,15]. In Section 5.1, we detail the source and process of the data. In Section 5.3 we show our experimental results in detail. The algorithm was coded in Python and tested on a PC with Intel Core i7 CPU.

### 5.1. Data Generation

The dataset used in the experiment was generated based on agricultural plant protection in the real world. UAV parameter and agricultural plant protection task information were in line with the agricultural plant protection in realworld.

#### 5.1.1. UAV Specifications

The UAV data came from the MG-1P model agricultural plant protection UAV produced by Dajiang Science and Technology [44]. According to its technical specifications, the agricultural plant protection UAV parameter settings can be seen as shown Table 4.

From Table 4 and the actual situation, we can know that energy costs 25 Ah–26 Ah per hour, and we conclude that the UAV can work for about 30 min after each energy replenishment. In addition, in order to accurately simulate the information of the action, we also need to get the relevant specifications of the plant protection UAV operation. Table 5 shows the MG-1P model agricultural plant protection UAV’s specifications in operation.

From Table 5, we can know that the maximum operating area of plant protection UAV is about 3000 square meters per minute and the flying speed of UAV is 12 m per second when not operating.

#### 5.1.2. Farm Information

In order to make the simulated task information more in line with the actual situation, we need to build simulation data with real farms as the background. In this work, we surveyed a farm in Quanzhou, Fujian, China [45].

This farm grows rare edible mushrooms such as mushrooms, straw mushrooms, tea tree mushrooms, comatus comatus, and pleurotus eryngii, and has 800 fields for growing mushrooms, with a total planting area of about 400,000 square meters [45]. In order to prevent pests, some crops need to be sprayed twice with pesticides.

#### 5.1.3. Data Simulation

According to Section 5.1.1, the maximum operating area of plant protection UAV is about 3000 square meters per minute and the flying speed of a UAV is 12 m per second when not operating. Here we set each action to 10 s, so each action *f* moves forward 120 m, each action *b* moves backward 120 m, each action *l* moves left 120 m, each action *r* moves right 120 m, and each action *p* has a work area of 500 square meters. The maximum working time of the UAV after each supplement of energy is set to 30 min.

According to Section 5.1.2, the average area of each field is 500 square meters, so we randomly generate an area of field by uniform distribution between 250–750. The distance between the base station and the field and the distance between fields are randomly generated by a uniform distribution between 300–900, so in order to prevent the UAV from running out of energy before returning to the base station, it needs to reserve enough energy to cost 75 s to ensure that it can return to the base station. The UAV was allocated 80 plant protection tasks randomly. It is worth noting that operations that can be completed in less than one action are still calculated as one action.

### 5.2. Evaluation Indicator

We use three evaluation indicators to evaluate the algorithm we proposed: Total score, total steps, and average score.

The total score is calculated according to the Definition 10, the formal definition in the following.

**Definition** **11.** *(Total Score)*

$$TS=\sum_{initial}^{termination}E\left(s,a\right).$$

In general, one point will be deducted for each wrong choice of action, but it is worth noting that some actions that are wrongly selected can cause great damage. For instance, if the UAV does not choose to supplement energy when the energy is insufficient, it may cause damage to the UAV. In order to distinguish this wrongly chosen action from other wrongly chosen actions, we set that 50 points will be deducted from the total score if the UAV does not choose to supplement energy when the energy is insufficient and one point will be deducted from the total score if other incorrect actions are chosen.

As the total score is not enough to measure the optimality of the policy, another evaluation indicator to assist measurement is total steps, that is the total number of actions executed by the UAV from start to completion.

The average score is the total score divided by total steps from start to completion. However it should be noted that when the total score is less than 0, the average score is meaningless, and only when the total score is greater than 0, the average score can reflect the optimality of the policy.

### 5.3. Result

**Figure 1:**
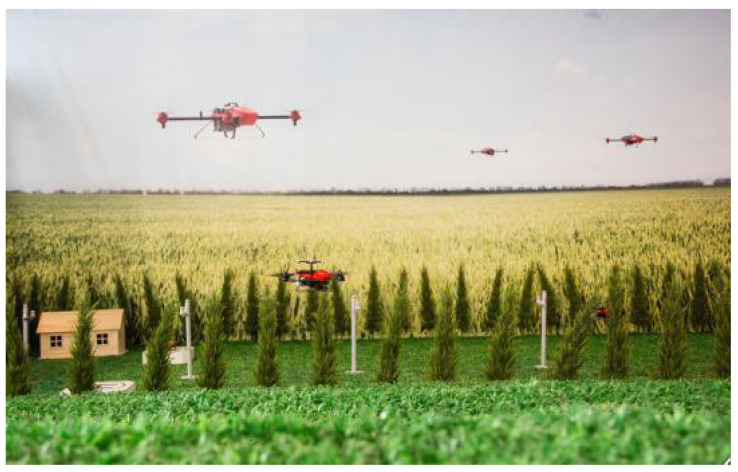
UAVs’scenario in an agricultural plant protection environment.

In this section, we provide details of the experimental performance of Approximate State Matching (ASM) Q-learning algorithm. The scene figure which represents the UAVs’scenario in an agricultural plant protection environment is shown in Figure 1 [46]. As shown in Section 5.2, we will use the total score and total steps as indicators to evalaute the algorithm. We will show the change curve of these two indicators as the number of episodes increases, the episode represents the agent executes a policy in the environment from beginning to end. Since too many steps were executed in the initial stage in the environment of agricultural plant protection, we set two conditions for entering the next episode: (1) The UAV completes all allocated agricultural plant protection tasks and (2) the total number of steps exceeds 6000. The ϵ−greedy policy is used to choose actions and the ϵ is set to 0.9. To better illustrate the superiority of our algorithm, we compare our proposed algorithm with the classic Q-learning algorithm and Deep Reinforcement Learning algorithm (DRL).

The steps of the classic Q-learning algorithm can be found in Algorithm 1. The DRL algorithm takes the state as input and calculates through the neural network to get the selected action. In this work, the network structure used is Long Short-term Memory (LSTM) which is a special type of Recurrent Neural Network (RNN) and proposed in [39]. The authors in [5] used LSTM to solve the problem of avoiding moving obstacles.

**Figure 2:**
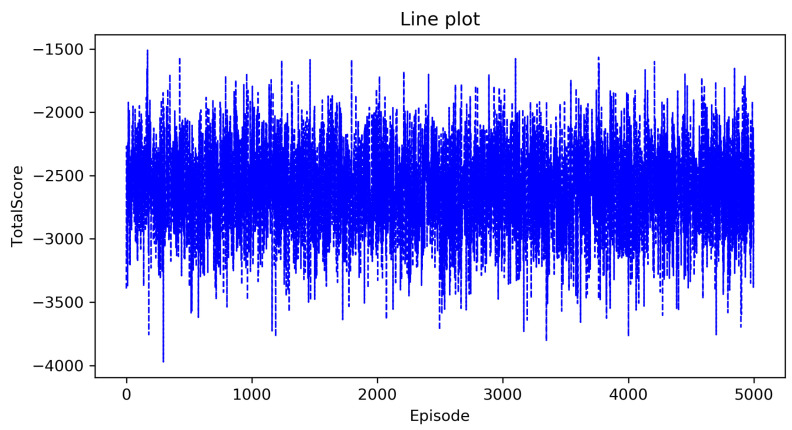
The total score of the classic Q-learning algorithm.

**Figure 3:**
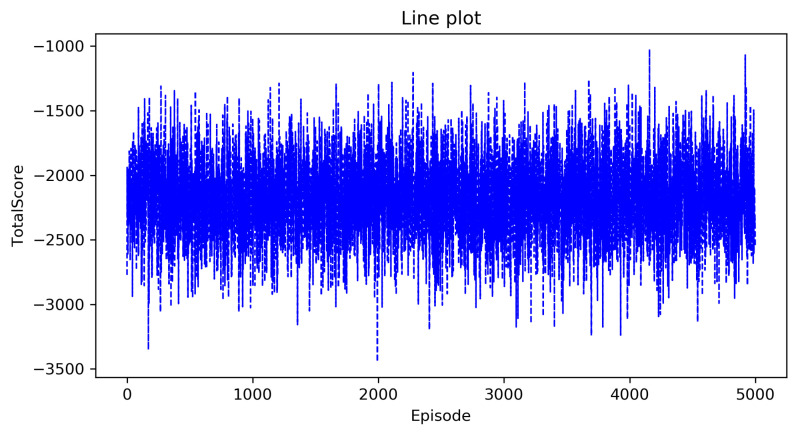
The total score of the DRL algorithm.

Figure 2 and Figure 3 show the curve of the total score as the number of episodes increases in the classic Q-learning algorithm and the DRL algorithm, the horizontal axis is the number of episodes that represents that the agent executes a policy in the environment from beginning to end, and the vertical axis is the total score. From these two figures, we can see that as the number of episodes increases, the total score has been oscillating at a low value, which indicates that the complete optimal policy has not been formed. This is consistent with our previous analysis. As the state space is too large, these two algorithms cannot solve the decision-making problem in the agricultural plant protection environment.

**Figure 4:**
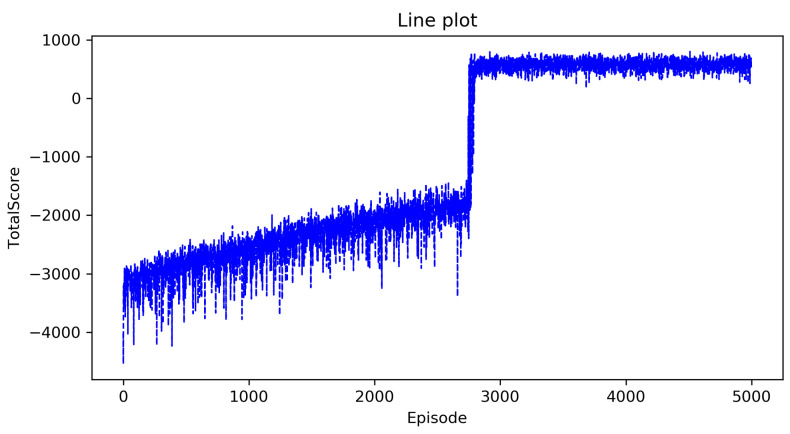
The ASM Q-learning algorithm when the maximum number of steps does not exceed 6000.

**Figure 5:**
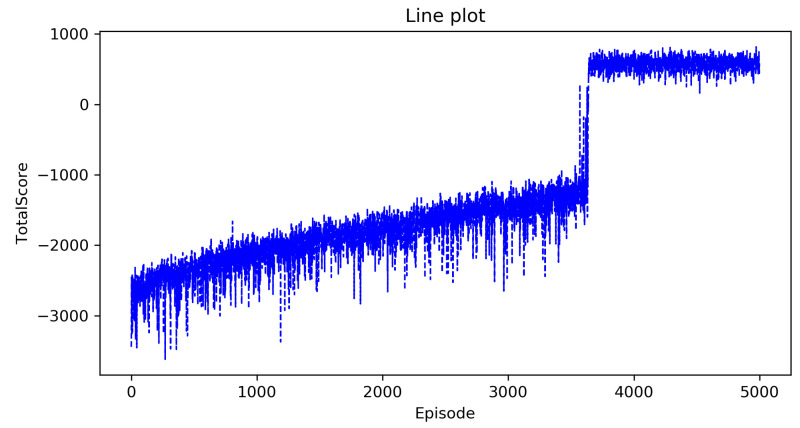
The ASM Q-learning algorithm when the maximum number of steps does not exceed 5000.

We first need to explore the impact of the limitation on the maximum number of total steps on the results. Figure 4 shows the curve of the total score as the number of episodes increases when the maximum number of total steps is set to 6000 and Figure 5 shows the curve of the total score as the number of episodes increases when the maximum number of total steps is set to 5000. From the two figures, we can see that the maximum number of total steps affects the speed of convergence, but has no effect on the total score. This is understandable, because the total number of steps needed to form a complete policy is roughly the same, so the maximum number of steps per episode is inversely proportional to the number of episodes. However it has little effect on the total score and the running time of the algorithm. Thus, next we will only study a situation where the maximum number of total steps is set to 6000.

Figure 4 shows the curve of the total score as the number of episodes increases in the Approximate State Matching Q-learning algorithm. In Figure 4, the horizontal axis is the number of episodes that represents the agent executing a policy in the environment from beginning to end, and the vertical axis is the total score.

We can see that the total score rises from a very low value in the first 2900 episodes, this is because: (1) The actions are randomly selected at the begining, so there are more incorrect choices than correct choices and (2) the policy is gradually updated to optimal but the complete policy has not yet been formed since the total number of steps is limited to 6000.

Between about 2900–3000 episodes, the total score has increased rapidly, which means that the complete policy has been initially formed, and the obvious wrong action selection of the UAV has been avoided.

After 2800 episodes, the total score slowly increases in fluctuations because: (1) The algorithm randomly selects actions with a probability of 0.1 and (2) the action selection policy of the UAV has been further improved through the improvement of details.

**Figure 6:**
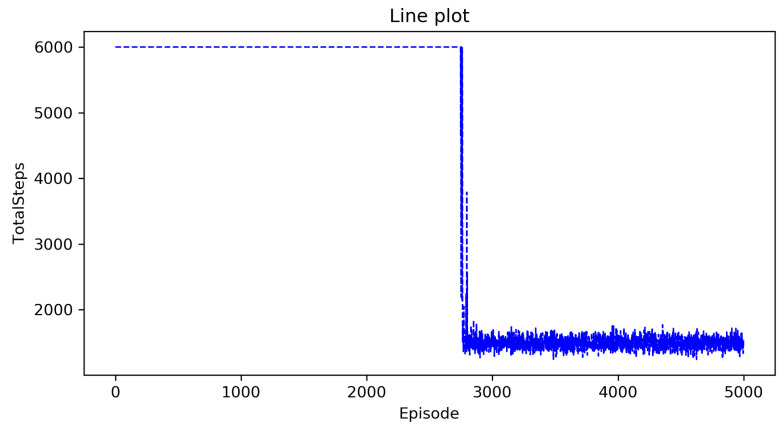
The total steps of the ASM Q-learning algorithm.

Figure 6 shows the curve of the total steps as the number of episodes increases in the Approximate State Matching Q-learning algorithm. In Figure 6, the horizontal axis is the number of episodes that represents the agent executing a policy in the environment from beginning to end, and the vertical axis is the total steps.

We can see that the total steps is fixed at 6000 in the first 2700 episodes, this is because the total steps that the UAV needs to complete all allocated agricultural plant protection tasks exceed 6000 at the begining due to the random selection of actions, so the total number of steps is 6000 according to the restrictions we made.

Between about 2750–2800 episodes, the total steps drop rapidly, which means that the complete policy has been initially formed, and the obvious wrong action selection of the UAV has been avoided.

After 2800 episodes, the total score slowly drops in fluctuations, this is because: (1) The algorithm randomly selects actions with a probability of 0.1 and (2) the action selection policy of the UAV has been further improved through the improvement of details.

**Figure 7:**
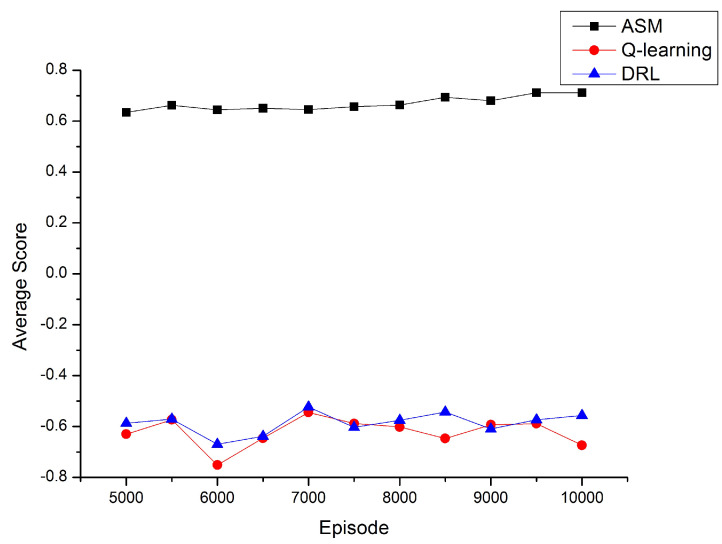
Comparison of average score of algorithms.

Figure 7 shows a comparison of the relationship between the average score and the number of episodes between different algorithms. From Figure 7, we can see that the average scores of the classical Q-learning algorithm and deep reinforcement learning algorithm have been fluctuating under the premise of being less than 0. This is because these two algorithms cannot get the optimal policy as the number of episodes increases, which is consistent with what is shown in Figure 2 and Figure 3. For the ASM Q-learning algorithm, we can see that as the number of episodes increases from 5000 to 10,000, the average score is gradually increasing. Theoretically, the optimal value of the average score is 1, but considering the strict deduction we set that 50 points will be deducted from the total score if the UAV does not choose to supplement energy when the energy is insufficient and one point will be deducted from the total score if other incorrect actions are chosen, this result will be a good performance. In Section 4.2, we have proven that incorrect actions can be avoided when selecting actions by referring to similar states in the past. However in practice, in order to reduce the time complexity of the algorithm, the past state we randomly explored no more than 200, so in some special cases, the wrong action may still be selected, so this affects the performance of the average score.

## 6. Conclusions and Future Work

In this work, to solve the decision-making support problem in the agricultural plant protection environment, an UAV agricultural plant protection model is established. We proposed an Approximate State Matching Q-learning algorithm which can obtain the optimal policy for UAVs. We analyzed the performance of the proposed algorithm and proved its advantages over the classic Q-learning algorithm in the agricultural plant protection environment through theorems. Experiments validated our analysis and we compared our proposed method with the classic Q-learning algorithm and DRL algorithm. The obtained results showed that the classic Q-learning algorithm and deep reinforcement learning algorithm were not suitable for solving policy learning in the agricultural plant protection environment while the algorithm we proposed could achieve a good performance.

In future work, we will improve our allocation algorithm to adapt to more complex realities, such as the consideration of cooperation between multiple UAVs in an agricultural plant protection environment. We will try to improve traditional RL algorithms or DRL to solve similar and more complex problems.

## Figures and Tables

**Table 1:** Implication of symbols.

Symbols	Implication
$S_{u}$	State collection of UAVs in an agricultural plant protection environment.
$A_{u}$	Action collection of UAVs in a agricultural plant protection environment.
$T_{u}$	State transition function in a plant protection environment.
$R_{u}$	Reward function in a plant protection environment.
len(stack)	Number of states in the stack.
Q(s,a)	The *Q* value of action *a* in state *s*.
Q(s,temporary)	The temporary *Q* value is used to assist the selection of action.
$S_{vp}$	Collection of states with similar positions.
$S_{ene}$	Collection of states with similar energy.

**Table 2:** Implication of actions.

Action	Implication
Forward	UAV goes forward one step, the distance of the step is fixed. Each Forward action performed costs 1 unit of energy.
Back	UAV goes back one step, the step of go back is the same as the step of go forward. Each Back action performed costs 1 unit of energy, which is the same as the energy cost of going Forward.
Left	UAV goes left one step, the step of go back is the same as the step of go forward. Each Back action performed costs 1 unit of energy, which is the same as the energy cost going Forward.
Right	UAV goes right one step, the step of go back is the same as the step of go forward. Each Back action performed costs 1 unit of energy, which is the same as the energy cost of going Forward.
Spray Pesticides	The UAV sprays pesticides once and costs 1 unit of energy, which is the same as the energy cost going Forward and Back.
Supplement Energy	The UAV returns to the base station and replenishes energy.

**Table 3:** *Q* table.

State	*f*	*b*	*p*	*e*
$s_{1}$	0	0	0	0
$s_{2}$	0.1	0	0	0
$s_{3}$	0	0	0.1	0
$s_{4}$	−0.1	−0.1	−0.1	0
⋯	⋯	⋯	⋯	⋯

**Table 4:** UAV specifications.

Model	MG-12000P MAH-44.4 V
Capacity	12,000 mAh
Compatible Aircraft Models	DJI MG-1P
Voltage	44.4 V
Battery Type	LiPo 12S
Energy	532 Wh
Net Weight	4.0 kg
Max Charging Power	1200 W

**Table 5:** Operating specifications.

Control range	3000 m
Work Coverage Width	4–7 m
Working Speed	7 m/s
Flight Speed	12 m/s

## Data Availability

Data is contained within the article, the data are presented in the Experiment of this study.

## References

[1] Watkins C.J.C.H., Dayan P. (1992). Q-learning. Mach. Learn..

[2] Peters J. (2010). Policy gradient methods. Scholarpedia.

[3] Zhang G., Li Y., Xu X., Dai H. (2019). Efficient Training Techniques for Multi-Agent Reinforcement Learning in Combat Tasks. IEEE Access.

[4] Khriji L., Touati F., Benhmed K., Al-Yahmedi A. (2011). Mobile robot Navigation Based on Q-Learning Technique. Int. J. Adv. Robot. Syst..

[5] Sun L., Zhai J., Qin W. (2019). Crowd Navigation in an Unknown and Dynamic Environment Based on Deep Reinforcement Learning. IEEE Access.

[6] Nguyen H., La H. Review of Deep Reinforcement Learning for Robot Manipulation. Proceedings of the 2019 Third IEEE International Conference on Robotic Computing (IRC).

[7] Jeyaratnam J. (1990). Acute pesticide poisoning: A major global health problem. World Health Stat. Q..

[8] Sun F., Wang X., Zhang R. (2020). Fair Task Allocation When Cost of Task Is Multidimensional. Appl. Sci..

[9] Wang G., Han Y., Li X., Andaloro J., Lan Y. (2020). Field evaluation of spray drift and environmental impact using an agricultural unmanned aerial vehicle (UAV) sprayer. Sci. Total Environ..

[10] Wang G., Yubin L., Qi H., Chen P., Hewitt A.J., Han Y. (2019). Field evaluation of an unmanned aerial vehicle (UAV) sprayer: Effect of spray volume on deposition and the control of pests and disease in wheat. Pest Manag. Sci..

[11] Ajmeri N., Guo H., Murukannaiah P.K., Singh M.P. Robust Norm Emergence by Revealing and Reasoning about Context: Socially Intelligent Agents for Enhancing Privacy. Proceedings of the Twenty-Seventh International Joint Conference on Artificial Intelligence.

[12] Hao J., Leung H.F. The dynamics of reinforcement social learning in cooperative multiagent systems. Proceedings of the Twenty-Third International Joint Conference on Artificial Intelligence (IJCAI ’13).

[13] Bo X., Liping C., Yu T. (2015). Path planning Based on Minimum Enerny Consumption for plant Protection UAVs in Sorties. Trans. Chin. Soc. Agric. Mach..

[14] Wang Y., Chen H., Li Y., Li H. (2017). Path Planning Method Based on Grid-GSA for Plant Protection UAV. Trans. Chin. Soc. Agric. Mach..

[15] Wang Y., Chen H., Li H. (2018). 3D Path Planning Approach Based on Gravitational Search Algorithm for Sprayer UAV. Trans. Chin. Soc. Agric. Mach..

[16] Sun F., Wang X., Zhang R. (2020). Task scheduling system for UAV operations in agricultural plant protection environment. J. Ambient. Intell. Humaniz. Comput..

[17] Tang J. (2019). Conflict Detection and Resolution for Civil Aviation: A Literature Survey. IEEE Aerosp. Electron. Syst. Mag..

[18] Tang J., Zhu F., Piera M.A. (2018). A causal encounter model of traffic collision avoidance system operations for safety assessment and advisory optimization in high-density airspace. Transp. Res. Part C Emerg. Technol..

[19] Conte R., Dignum F. (2001). From Social Monitoring to Normative Influence. J. Artif. Soc. Soc. Simul..

[20] Alechina N., Dastani M., Logan B. Programming norm-aware agents. Proceedings of the 11th International Conference on Autonomous Agents and Multiagent Systems—Volume 2.

[21] Gasparini L., Norman T.J., Kollingbaum M.J. (2018). Severity-sensitive norm-governed multi-agent planning. Auton. Agents Multi-Agent Syst..

[22] Meneguzzi F., Luck M. Norm-based behaviour modification in BDI agents. Proceedings of the 8th International Conference on Autonomous Agents and Multiagent Systems (AAMAS ’09)—Volume 1.

[23] Dignum F., Morley D., Sonenberg E., Cavedon L. (2000). Towards socially sophisticated BDI agents. Proceedings of the Fourth International Conference on MultiAgent Systems.

[24] Fagundes M.S., Billhardt H., Ossowski S. Normative reasoning with an adaptive self-interested agent model based on Markov decision processes. Proceedings of the 12th Ibero-American Conference on Advances in Artificial Intelligence (IBERAMIA’10).

[25] Ajmeri N., Jiang J., Chirkova R., Doyle J., Singh M.P. Coco: Runtime reasoning about conflicting commitments. Proceedings of the Twenty-Fifth International Joint Conference on Artificial Intelligence (IJCAI’16).

[26] van Riemsdijk M.B., Dennis L., Fisher M., Hindriks K.V. A Semantic Framework for Socially Adaptive Agents: Towards strong norm compliance. Proceedings of the 2015 International Conference on Autonomous Agents and Multiagent Systems (AAMAS ’15).

[27] Watkins C.J.C.H. (1989). Learning from Delayed Rewards. Ph.D. Thesis.

[28] Smart W., Kaelbling L.P. Effective reinforcement learning for mobile robots. Proceedings of the 2002 IEEE International Conference on Robotics and Automation (Cat. No.02CH37292).

[29] Martinez-Gil F., Lozano M., Fernández F. Multi-agent reinforcement learning for simulating pedestrian navigation. Proceedings of the 11th International Conference on Adaptive and Learning Agents.

[30] Casadiego L., Pelechano N. From One to Many: Simulating Groups of Agents with Reinforcement Learning Controllers. Proceedings of the Intelligent Virtual Agents: 15th International Conference (IVA 2015).

[31] Li S., Xu X., Zuo L. Dynamic path planning of a mobile robot with improved Q-learning algorithm. Proceedings of the 2015 IEEE International Conference on Information and Automation.

[32] Bianchi R.A.C., Ribeiro C.H.C., Costa A.H.R. (2004). Heuristically Accelerated Q–Learning: A New Approach to Speed Up Reinforcement Learning. Proceedings of the Brazilian Symposium on Artificial Intelligence.

[33] Matignon L., Laurent G., Piat N.L.F. (2008). A study of FMQ heuristic in cooperative multi-agent games. Proceedings of the 7th International Conference on Autonomous Agents and Multiagent Systems. Workshop 10: Multi-Agent Sequential Decision Making in Uncertain Multi-Agent Domains, Aamas’08.

[34] Ng A.Y., Russell S.J. (2000). Algorithms for Inverse Reinforcement Learning. Proceedings of the Seventeenth International Conference on Machine Learning.

[35] Henry P., Vollmer C., Ferris B., Fox D. Learning to navigate through crowded environments. Proceedings of the 2010 IEEE International Conference on Robotics and Automation.

[36] Anschel O., Baram N., Shimkin N. Averaged-DQN: Variance reduction and stabilization for deep reinforcement learning. Proceedings of the 34th International Conference on Machine Learning—Volume 70.

[37] Wang P., Li H., Chan C.Y. Continuous Control for Automated Lane Change Behavior Based on Deep Deterministic Policy Gradient Algorithm. Proceedings of the 2019 IEEE Intelligent Vehicles Symposium (IV).

[38] Schulman J., Wolski F., Dhariwal P., Radford A., Klimov O. (2017). Proximal Policy Optimization Algorithms. arXiv.

[39] Hochreiter S., Schmidhuber J. (1997). Long Short-Term Memory. Neural Comput..

[40] Zhang Y., Cai P., Pan C., Zhang S. (2019). Multi-Agent Deep Reinforcement Learning-Based Cooperative Spectrum Sensing With Upper Confidence Bound Exploration. IEEE Access.

[41] Matta M., Cardarilli G.C., Nunzio L.D., Fazzolari R., Giardino D., Nannarelli A., Re M., Spanò S. (2019). A Reinforcement Learning-Based QAM/PSK Symbol Synchronizer. IEEE Access.

[42] Littman M.L., Szepesvári C. A Generalized Reinforcement-Learning Model: Convergence and Applications. Proceedings of the Machine Learning, Thirteenth International Conference (ICML ’96).

[43] Kaelbling L.P., Littman M.L., Moore A.W. (1996). Reinforcement learning: A survey. J. Artif. Intell. Res..

[44] Science D. MG-1200P Flight Battery User Guide. https://dl.djicdn.com/downloads/mg_1p/20180705/MG-12000P+Flight+Battery+User+Guide_Multi.pdf.

[45] Cai P., Lin M., Huang A. (2012). Research on the Informatization Construction of Agricultural Cooperatives—A Case Study of Rural Areas in Southern Fujian. J. Jiangxi Agric..

[46] News N. Japanese Companies Develop New UAVs to Cope with Aging Farmers. https://news.163.com/air/18/0904/15/DQSCN107000181O6.html.

